# Doublecortin (DCX) is not Essential for Survival and Differentiation of Newborn Neurons in the Adult Mouse Dentate Gyrus

**DOI:** 10.3389/fnins.2015.00494

**Published:** 2016-01-11

**Authors:** Jagroop Dhaliwal, Yanwei Xi, Elodie Bruel-Jungerman, Johanne Germain, Fiona Francis, Diane C. Lagace

**Affiliations:** ^1^Neuroscience Program, Department of Cellular and Molecular Medicine, University of OttawaOttawa, ON, Canada; ^2^Institut National de la Santé et de la Recherche Médicale UMRS 839Paris, France; ^3^Sorbonne Universités, Université Pierre et Marie CurieParis, France; ^4^Institut du Fer à MoulinParis, France; ^5^Institut National de la Santé et de la Recherche Médicale UMRS 952Paris, France

**Keywords:** doublecortin, adult neurogenesis, DCX knockdown, DCX knockout, survival, differentiation, retrovirus labeling

## Abstract

In the adult brain, expression of the microtubule-associated protein Doublecortin (DCX) is associated with neural progenitor cells (NPCs) that give rise to new neurons in the dentate gyrus. Many studies quantify the number of DCX-expressing cells as a proxy for the level of adult neurogenesis, yet no study has determined the effect of removing DCX from adult hippocampal NPCs. Here, we use a retroviral and inducible mouse transgenic approach to either knockdown or knockout DCX from adult NPCs in the dentate gyrus and examine how this affects cell survival and neuronal maturation. Our results demonstrate that shRNA-mediated knockdown of DCX or Cre-mediated recombination in floxed DCX mice does not alter hippocampal neurogenesis and does not change the neuronal fate of the NPCs. Together these findings show that the survival and maturation of adult-generated hippocampal neurons does not require DCX.

## Introduction

The microtubule-associated protein Doublecortin (DCX) is widely expressed in developing neurons during embryonic and early postnatal development throughout the central and peripheral nervous system (Francis et al., 1999; Gleeson et al., 1999; Belvindrah et al., 2011). In the adult brain the expression of DCX is restricted to the neurogenic regions, as well as specific non-neurogenic regions such as the piriform cortex (Bonfanti and Nacher, 2012). The changes in the expression profile of DCX throughout the lifespan, within both neurogenic and non-neurogenic cells, suggest that DCX may subserve multiple, different functions during development and in the adult, as well as within different cellular contexts during physiological and pathological states (Bonfanti and Nacher, 2012).

Within the two main adult neurogenic regions DCX is transiently expressed in the dividing neuronal precursor cells (NPCs) until the cells become mature neurons after approximately 30 days (Brown et al., 2003). Within the neurogenic region of the adult rostral migratory stream (RMS), where NPCs migrate from the subventricular zone (SVZ) to the olfactory bulb (OB), the function of DCX has been explored. In young adult DCX mutant mice, DCX was first shown to be important for the maintenance of their bipolar shape, migration and fate (Koizumi et al., 2006b). Work in early postnatal mice has also highlighted a novel role for DCX in determining the fate of the neurogenic cells in the RMS (Belvindrah et al., 2011). Yet in direct contrast to these findings, Merz and Lie (2013) recently found no migration or fate phenotype when using a microRNA mediated retroviral approach to knockdown DCX from the RMS in adult mice. These conflicting findings suggest that DCX may have regional and age-dependent roles within the SVZ-RMS-OB.

Within the adult neurogenic region of the dentate gyrus only one study has tested the cell-intrinsic role of DCX. Merz and Lie (2013) found no hippocampal neurogenic deficits following their microRNA mediated retroviral approach to knockdown DCX. However, as highlighted by Merz and Lie (2013), their conclusion may have been confounded by using a knockdown approach that preserved some DCX expression, as well as examining exercising adult mice. Thus, the effect of a complete removal of DCX specifically within adult NPCs that develop into granule neurons within the adult brain remains unknown. This study sought to elucidate the requirement of DCX in the formation of adult-generated granule neurons under normal physiological conditions. In the adult, we used retroviruses to target DCX-expressing NPCs to either (1) knockdown DCX using shRNA in C57Bl6 wild type mice, or (2) knockout DCX through expression of Cre in floxed DCX (DCX^flox^) mice. Additionally we examined DCX-null NPCs in a novel inducible transgenic mouse that allowed for the removal of DCX from the GLAST-expressing stem cells prior to expression of DCX. The data obtained from these multiple approaches provide convincing evidence that a reduction or removal of DCX is associated with no visible hippocampal neurogenic deficits in the adult.

## Materials and methods

### Animals

C57BL/6J mice were purchased from Charles River (female, 6–8 weeks). All procedures associated with the retroviral experiments were performed within the guidelines of the Canadian Council on Animal Care and were approved by the University of Ottawa Animal Care Committee. Generation of the floxed DCX mice has been previously described from a tri-loxP allele (Kappeler et al., 2006) and Cre recombined mice were produced, maintained and manipulated with authorization from the French Ministry of Research (00984.02). DCX is localized to the X-chromosome, thus males DCX^flox∕Y^ were compared to WT littermate controls. Glast-CreERT2 knockin mice, where the CreERT2 expression is driven by the sodium-dependent glutamate/aspartate transporter (Glast/Slc1a3), have been described previously (Mori et al., 2006). Mice were housed in a 12-h light/dark cycle with free access to food and water.

Adult GlastCreERT2 heterozygote x DCX flox hemizygote male mice on the 129 Sv/Pas genetic background were treated with tamoxifen (Sigma, France) dissolved in corn oil (Sigma, France), 10% ethanol. Two × 2.5 mg/day intraperitoneal injections were performed during 5 days, or corn oil (Vehicle) was injected alone (in 10% ethanol), as described previously (Mori et al., 2006). Four weeks after the last injection of tamoxifen, bromodeoxyuridine (BrdU, Fluka Analytical, Sigma-Aldrich Chemie Gmbh, Switzerland # 16880) was injected at 100 mg/kg (4X, each injection spaced by 2 h) in order to label dividing NPCs. Mice were sacrificed 10 days after BrdU injection.

### Retrovirus production and injection

The shDCX sequence (mature sense: CCCTATAGCTGTAGTTAGA) and scrambled control sequence (ATCTCGCTTGGGCGAGAGTAAG) were obtained from Dharmacon (#V2LHS_229318 and #RHS4346). The shDCX was verified to target both human and mouse DCX (nBLAST). The shDCX was further validated in the neuroblastoma cell line SHSY5Y (ATCC # CRL-2266) *in vitro* where it induced a significant 2.2 fold decrease in endogenous DCX protein vs. scrambled control. (0.552 ± 0.008 shDCX vs. 1.214 ± 0.164 control, normalized to tubulin, *p* < 0.05, *N* = 3).

For *in vivo* DCX knockdown, the shDCX or control sequences were cloned into a retroviral vector pUEG that co-express shDCX or scrambled control sequence under the U6 promoter and GFP under the EF1α promoter. The vector was generously provided by Dr. Hongjun Song (Johns Hopkins University).

For *in vivo* DCX knockout, the retroviral vectors CAG-GFP-Cre and the control CAG-RFP and the corresponding packing and envelope constructs were generously provided by Dr. Fred Gage (The Salk Institute). The retroviruses were generated as previously described and mixed in a 1:1 ratio prior to injection (Tashiro et al., 2006). The titers of viruses were determined by live titer using 293T cells and ranged between ~2 and 4 × 10^8^ infectious units (IU) per ml.

The retroviruses were bilaterally injected into the dentate gyrus of 6–8 week old mice. The shDCX or scrambled virus was injected into C57BL/6J mice. The mixture of CAG-GFP-Cre and CAG-RFP was injected into floxed DCX mice and littermate WT controls. The injections were performed using a Hamilton microsyringe and a 33 gauge needle into the dentate gyrus (−1.7 mm from Bregma, ±1.2 mediolateral, −2.4 mm dorsoventral; final volume: 1.5 μl per injection site, injection rate: 0.2 μl/min). Mice were kept under anesthetic via isoflurane during the surgery procedure and were allowed to recover in a warm chamber after surgery. The mice also received 3 subcutaneous injections of analgesic buprenorphine (0.05 mg/kg) 1 h prior to, as well as 5–6 h and 10–12 h after surgery.

### Tissue processing, immunohistochemistry, and quantification

Mice were anesthetized and transcardially perfused with cold phosphate buffered saline (1X PBS, pH 7.4) followed by 4% paraformaldehyde in 1x PBS (pH = 7.4). Brains were post-fixed for 1 or 24 h in 4% paraformaldehyde. All brains that were used for the retroviral studies following post-fix were transferred to 30% sucrose with 0.1% sodium azide in 1X PBS. Brains were cut coronally on a freezing microtome at 30 μm and stored in 1X PBS with 0.1% sodium azide. The GlastCreER^T2^ × floxDCX inducible mice brains were sectioned into 40 μm vibratome slices.

Immunohistochemistry was performed on free-floating sections. Sections were washed in 1X PBS and incubated in 0.1% Tween-20 and 0.1% Triton X-100 in 1X PBS with corresponding primary antibodies at 4°C overnight. The primary antibodies used were: chicken anti-GFP (GFP-1020, Aves, 1:5000), rabbit anti-dsRed (632496, Clontech, 1:5000), goat anti-DCX (sc-8066, Santa Cruz, 1:500), mouse anti-NeuN (MAB377, Millipore, 1:500), rat anti-BrdU (AbCys, France AbC117-7513, 1:1000) and goat anti-NeuroD1 (sc- 1084, Santa Cruz, 1:500). Sections were washed in 1X PBS, incubated with corresponding Cy2, Cy3, and Cy5-conjugated IgG secondary antibodies (Jackson Immuno, 1:500) for 1 h at room temperature, stained with DAPI (Roche, 1:10000) and washed in 1X PBS prior to mounting on slides and coverslipping.

Retroviral labeled cells in the SGZ were counted using an Olympus BX51 fluorescent microscope. The quantification of co-labeled cells was performed using a Zeiss LSM510 confocal microscope or Zeiss AxioObserver.Z1 microscope. For the GlastCreER^T2^ × floxDCX inducible mouse studies the BrdU and DCX labeled cells were counted manually using an apoptome microscope (Zeiss, Germany). All counts and phenotype analysis were performed at 400x magnification by an experimenter blind to the experimental conditions and genotypes.

### Statistical analysis

All data are reported as mean ± standard error of the mean (SEM) and statistical analysis was performed using GraphPad Prism (v6.0) software. An outlier test was performed on all retroviral counts and 3/86 counts from individual hippocampi were excluded. Experiments with two groups were analyzed by the two-tailed *Student t-test*. Analyses of two factors were performed using a Two-way ANOVA test followed by a Bonferroni *post hoc*. Statistical significance was defined as *p* < 0.05.

## Results

### Knockdown of DCX does not affect survival of adult-generated hippocampal neurons

To fate map the NPCs following either a knockdown or knockout of DCX *in vivo*, we utilized a retrovirus approach to infect and track the maturation of the dividing NPCs that develop into granule neurons, expressing NeuN by 30 days post infection (dpi). We and others have shown that this approach is effective at targeting the NPCs and immature neurons expressing DCX, with peak DCX expression found 3–14 dpi (Ge et al., 2006; Tashiro et al., 2006; Jessberger et al., 2008; Jagasia et al., 2009; Schnell et al., 2014; Ceizar et al., 2015).

**Figure 1:**
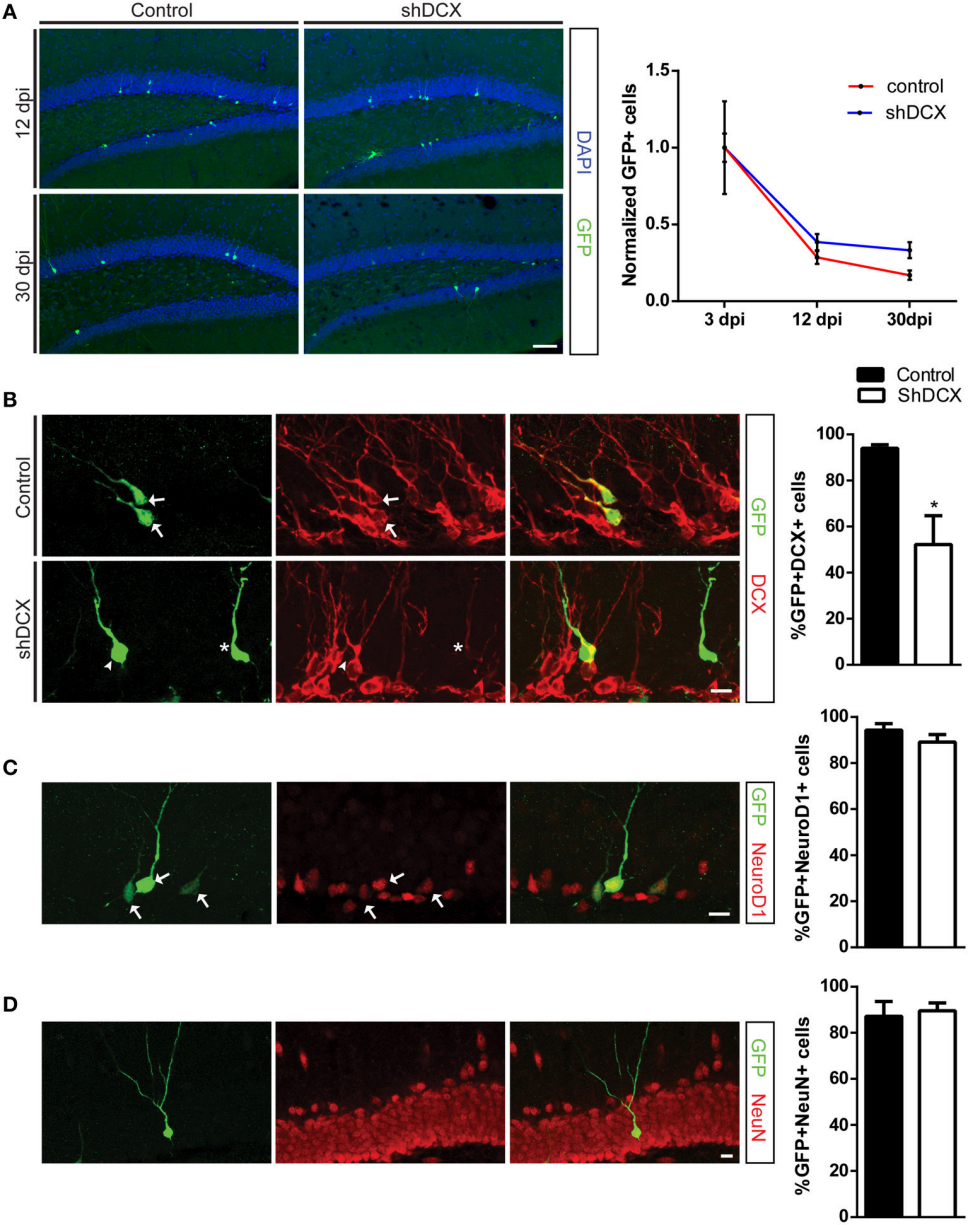
**Retroviral knockdown of DCX does not affect NPC survival and differentiation. (A)** Representative images and quantification of NPCs showing no difference in normalized counts between shDCX-GFP cells compared to Ctrl-GFP cells at 12 and 30 dpi; **(B)** Proportion of GFP+ NPCs expressing DCX was significantly reduced in shDCX-GFP compared to Ctrl-GFP, with some shDCX cells having fainter DCX staining (arrows = GFP+ DCX+; arrowhead = GFP+ DCX−; star = GFP+DCX+ faint). **(C)** Expression of the immature neuronal marker NeuroD1 is unaffected between control and shDCX expressing cells (arrows = GFP+NeuroD1+ colabeled cells). **(D)** Neuronal fate was not affected as there was no difference in proportion of GFP+ cells expressing the mature neuronal marker NeuN between control and shDCX at 30 dpi. *N* = 3–6 mice per group with the total number of cells analyzed in **(B)** 105 Ctrl and 127 shDCX cells; **(C)** 151 Ctrl and 201 shDCX cells; **(D)** 130 Ctrl and 84 shDCX cells. ^*^p < 0.05. Scale bar = 20 μm for **(A)** and 10 μm for **(B–D)**.

To knockdown DCX in the NPCs and their progeny, retroviruses expressing either shDCX-GFP or scrambled control (Ctrl-GFP) were created and stereotaxically injected into the dentate gyrus in C57BL/6 mice. We generated a time-course to examine and compare survival of Ctrl-GFP+ and ShDCX-GFP+ cells. There were significantly fewer shDCX-GFP+ cells at 3 and 12 dpi compared to Ctrl-GFP+ cells (Supplementary Figure 1). The reduction in number of shDCX-GFP cells at 3dpi was surprising and could be attributed to differences in *in vivo* viral transduction efficiency. In order to control for this variability and determine the survival rate ShDCX-GFP+ and Ctrl-GFP+ cells, the absolute cell numbers at 12 and 30 dpi were normalized to the average number of cells labeled at 3dpi in each group, as previously published also by others (Schnell et al., 2014). Analysis of the infected cells between 3 and 30 dpi demonstrated that relative to the number of cells present at 3 dpi both shDCX-GFP and Ctrl-GFP expressing cells, as expected, showed a decline in cell survival over time (Figure 1A). However, there was no significant difference in the relative survival of the cells between the shDCX-GFP+ and Ctrl-GFP+ at 12 and 30 dpi (Figure 1A).

To confirm that a knockdown of DCX had occurred *in vivo*, the percentage of shDCX-GFP and Ctrl-GFP expressing cells also expressing DCX was determined. There was a significant reduction in the percentage of the shDCX-GFP cells that expressed DCX compared to the control at 12 dpi (Figure 1B). Expression of DCX was observed in over 90% of control cells compared to only ~50% of shDCX-GFP infected cells. As shown in Figure 1B, the majority of the shDCX-GFP+ cells that showed DCX expression also had notably less bright DCX staining relative to the intensity of DCX observed in the Ctrl-GFP cells, suggestive of lower levels of DCX within these cells. In contrast to the significant reduction in percentage of cells expressing DCX, the proportion of surviving NPCs expressing the proneural basic helix-loop-helix transcription factor NeuroD1 was similar between shDCX-GFP and Ctrl-GFP infected cells at 12 dpi (Figure 1C). Furthermore, on average over 90% of surviving shDCX-GFP and Ctrl-GFP expressing cells at 30 dpi expressed the mature neuronal marker, NeuN (Figure 1D) suggesting DCX expression does not impact the neuronal fate of the dividing NPCs. Together these results show that reducing the amount of DCX in the NPCs does not affect the survival and neuronal fate of the cells during their development in the adult dentate gyrus.

### Knockout of DCX does not affect adult-generated hippocampal neurons

In order to completely remove DCX and track the development of the DCX-null NPCs we next used a retroviral method to express Cre in a floxed DCX mouse. A mixture of CAG-GFP-Cre and CAG-RFP was used to infect NPCs in both floxed DCX and WT mice. The CAG-RFP was used as a control virus to allow the survival ratio of the infected dividing cells to be calculated by comparing the ratio of double labeled (GFP+RFP+) to all control RFP+ NPCs, as we and others have previously published (Tashiro et al., 2006; Jessberger et al., 2008; Jagasia et al., 2009; Schnell et al., 2014; Ceizar et al., 2015).

**Figure 2:**
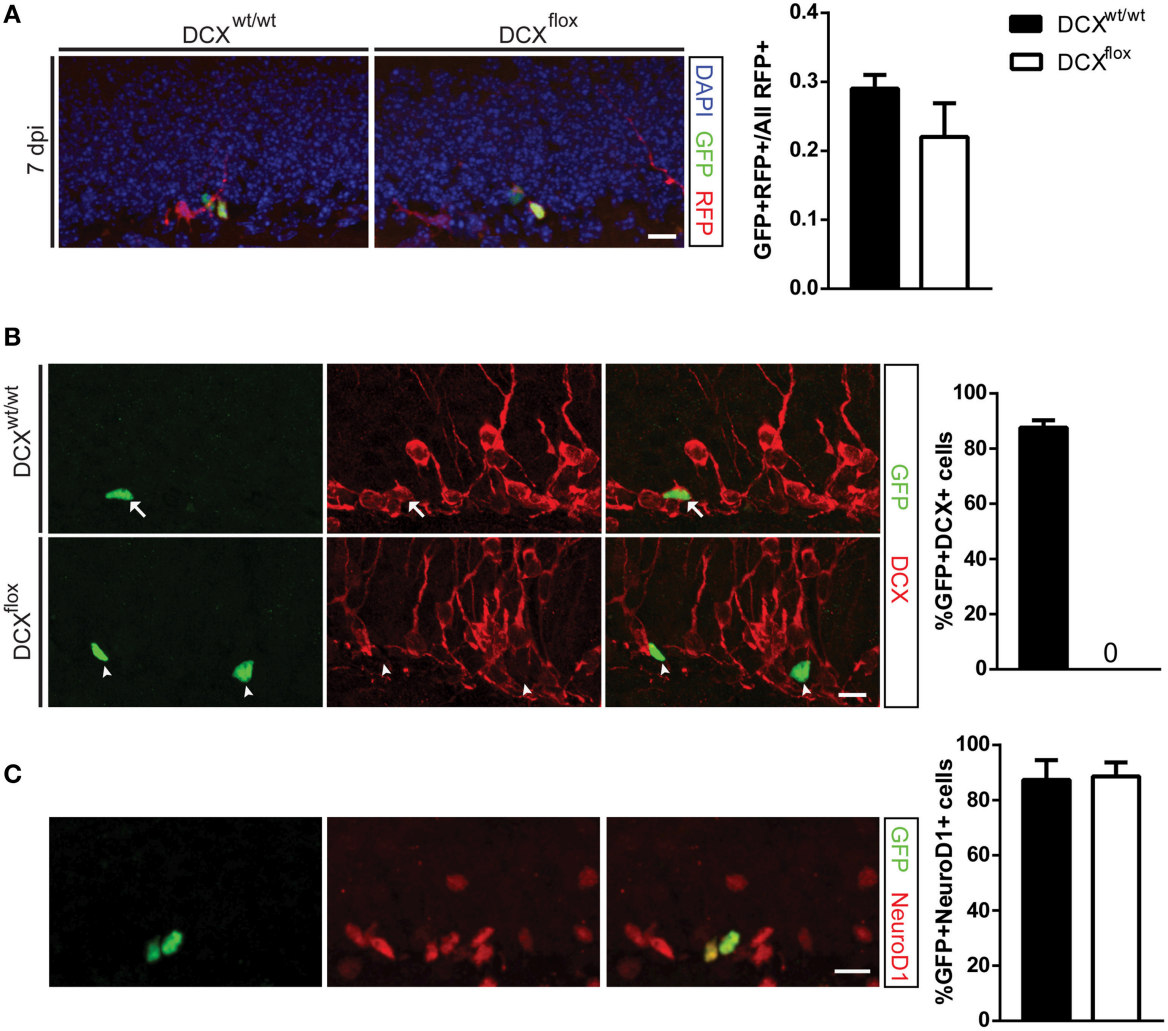
**Retroviral knockout of DCX abolishes DCX expression *in vivo* but does not affect cell survival at 7 dpi. (A)** Representative image and quantification showing the survival ratio of infected NPCs is the same in WT and floxed DCX (DCX^flox^) mice at 7 dpi. **(B)** Knockout of DCX was confirmed by no detectable GFP and DCX co-labeled cells in DCX^flox^ mice (arrows = GFP+DCX+ co-labeled cells, arrowhead = GFP+ DCX−). **(C)** Proportion of GFP-Cre NPCs expressing the immature marker NeuroD1 is similar between WT and DCX^flox^ mice. *N* = 3 mice per genotype with the total number of cells analyzed in **(B)** 113 WT cells and 111 KO cells; **(C)** 164 WT cells and 135 KO cells. Scale bars = 10 μm.

**Figure 3:**
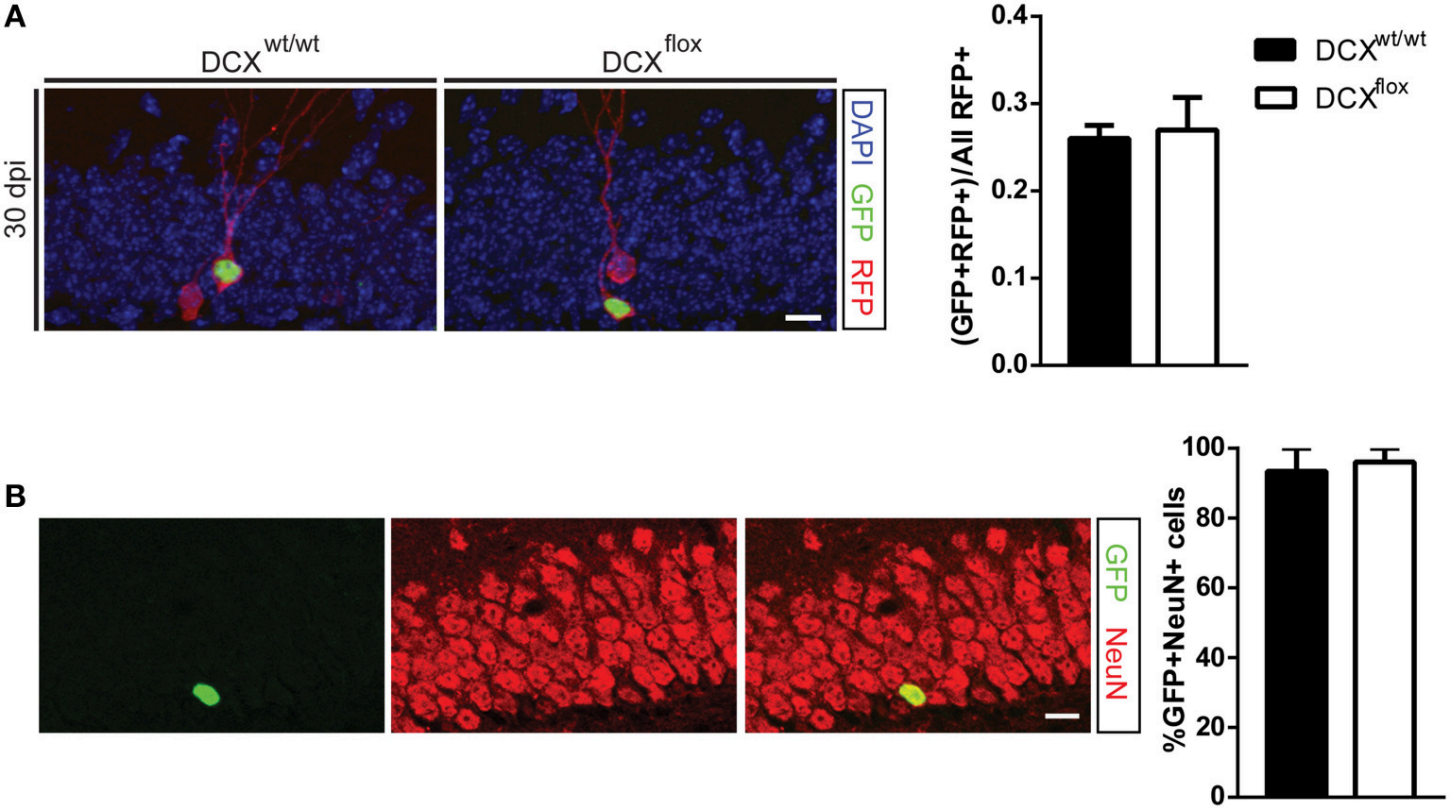
**Retroviral knockout of DCX does not affect the survival or fate of adult-generated neurons at 30 dpi. (A)** Representative image and quantification showing the survival ratio is the same in WT and floxed DCX (DCX^flox^) mice at 30 dpi. **(B)** Neuronal fate was not affected in DCX-null cells, with no difference in the proportion of GFP-labeled cells expressing the mature neuronal marker NeuN. *N* = 3–5 mice per genotype with the total number of cells analyzed in **(B)** 44 WT cells and 59 KO cells. Scale bars = 10 μm.

In agreement with the shDCX results, the survival of DCX-null vs. DCX-expressing infected cells was comparable at 7 dpi (Figure 2A). This result was not attributable to low efficiency of the GFP-Cre, as all GFP-Cre cells in the floxed DCX mice were absent of DCX expression (Figure 2B), yet similar to the shDCX-GFP cells, DCX-null cells expressed NeuroD1 (Figure 2C). Similarly, at 30 dpi there was no change in survival of DCX-null cells (Figure 3A). DCX also appeared not to be essential for neuronal maturation and fate as nearly all DCX-null and DCX-expressing cells expressed NeuN (Figure 3B).

**Figure 4:**
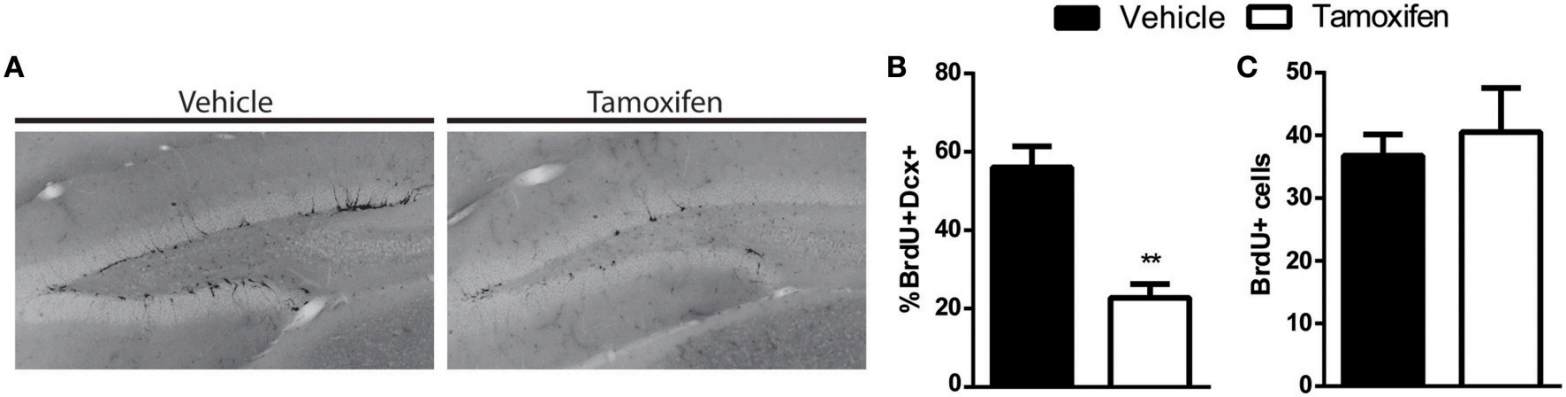
**GlastCreER^T2^ x floxDCX inducible knockout of DCX does not impact survival of dividing NPCs. (A)** Representative image showing less DCX+ cells in the knockout mice 4 weeks after tamoxifen treatment. **(B)** The proportion of cells expressing both BrdU and DCX is significantly reduced in tamoxifen treated group. **(C)** The overall number of 10-day old BrdU+ cells is similar between the inducible knockout mice and control. *N* = 4 mice per group. ^**^p < 0.005.

By using a retroviral approach to knockout DCX we cannot exclude the possibility that DCX was expressed in the NPCs before the knockout, since retroviruses infect dividing cells, including ones that already express DCX (Ge et al., 2006; Ceizar et al., 2015). This raises the possibility that the lack of phenotype in the DCX-null cells may be in some cases attributed to prior DCX expression. In order to address this possibility, we also created an inducible GlastCreER^T2^ x floxed DCX mouse to test if removal of DCX prior to DCX expression in NPCs altered the phenotype of the maturing NPCs. In this inducible mouse, tamoxifen was injected in order to prevent DCX expression from the Glast-expressing neural stem cells. At 4 weeks after tamoxifen injection the mice were given a pulse of BrdU to label the NPCs and mice were sacrificed 10 days later, a time point when there is a significant proportion of immature cells that express DCX (Ninkovic et al., 2007). As expected there was a reduction in the total number of DCX cells in the tamoxifen treated GlastCreER^T2^ × floxed DCX mice (Figure 4A). To quantify the reduction, the proportion of BrdU+ NPCs expressing DCX was examined 10 days after BrdU labeling. As expected, there was a significant reduction of DCX+ cells in the tamoxifen-injected floxed mice (Figure 4B). Although less BrdU+ cells expressed DCX, the total number of BrdU+ cells was similar between the vehicle and tamoxifen treated groups (Figure 4C). This data therefore supports our combined retroviral data which together suggest that knockout of DCX does not affect the survival of the developing NPCs in the adult hippocampus.

## Discussion

Our findings show that either reducing DCX expression by the retroviral shRNA knockdown approach or knocking out DCX by retroviral delivery of Cre does not alter the survival of adult-generated neurons. We confirm using the GlastCreER^T2^ × floxDCX inducible mouse that NPC survival is unaffected even when the knockout is initiated in the GLAST-expressing neural stem cells, which ultimately give rise to the DCX expressing progeny. Additionally we find knocking down or removing DCX does not change the neuronal fate of the maturing NPCs.

Our data is in agreement with recent results obtained in a constitutive DCX knockout mouse studied by Germain et al. (2013) and after a knockdown of DCX using micro-RNA in neurogenic cells by Merz and Lie (2013). The DCX knockout mice showed an absence of DCX and no changes in adult hippocampal neurogenesis. Since the KO of DCX was constitutive, the lack of phenotype could be attributed to compensatory mechanisms, as have been previously identified by differences observed between embryonic and adult-specific knockout models (Urbán and Guillemot, 2015). However, Merz and Lie (2013) also identified no significant effects on adult neurogenesis following reducing the levels of DCX specifically in the adult dividing NPCs using a micro-RNA knockdown approach. The lack of deficits following knockdown of DCX however can be confounded by the preservation of some DCX protein expression. Therefore, to more extensively examine the function of DCX in the dentate NPCs we used a retroviral and inducible transgenic mouse approach to knockout DCX from the NPCs. In this case, the DCX-null NPCs showed similar survival compared to WT. Thus, unlike the regional and age-dependent roles of DCX proposed within the SVZ-RMS-OB, within the neurogenic cells of the dentate DCX does not appear to be required for cell survival.

Both the shDCX-GFP and DCX-null NPCs generated NeuN+ mature granule neurons suggesting that DCX is also not essential for neuronal fate commitment, *per se*, during adult neurogenesis. The lack of requirement for DCX for neuronal differentiation was also supported by Merz and Lie (2013) who found that a reduction in DCX within NPCs did not affect the fate of NPCs in mice that had free access to running wheels. Our work extends this study by showing DCX is dispensable for neuronal fate during basal hippocampal neurogenesis, which is notable given that running may enhance the expression of factors that could render the newly generated neurons resistant to loss of DCX. Furthermore, the role of DCX in modulating differentiation is likely cell-type specific. For example, DCX is a differentiation-based treatment *in vivo* for glioma, where DCX induces terminal differentiation of glioma cells via a DCX/GFAP (glial fibrillary acidic protein) pathway (Santra et al., 2010).

Our results showing no hippocampal neurogenic deficits from both DCX knockdown and knockout approaches in the adult NPCs is also notable given a variety of studies have reported conflicting results between DCX knockdown and knockout approaches. This is especially well-documented in studies examining the functional role of DCX in neocortical development, where shRNA-mediated knockdown of DCX results in deficits, whereas germ line DCX knockout models or micro-RNA mediated DCX knockdown show no neocortical deficits (Corbo et al., 2002; Bai et al., 2003; Götz, 2003; Baek et al., 2014). Recently this conflicting data has been resolved through the discovery that the neocortical deficits associated with the shRNA-mediated knockdown of DCX can be attributed to off-target effects associated with noncoding microRNAs (Baek et al., 2014). In our analysis of adult shRNA knockdown of DCX, the survival of Ctrl-GFP+ and shDCX-GFP+ cells was calculated by normalizing absolute cell numbers to the average number of cells labeled at 3dpi since there were significantly fewer shDCX-GFP+ cells at 3 dpi compared to Ctrl-GFP+ cells. The reduction in number of shDCX-GFP cells at this early time point we hypothesized to be attributed to differences in viral transduction efficiency, despite using similar amounts and viral titers for infection. It is unlikely that this effect is due to the off-target effects of DCX family shRNA, given that off-target miRNA effects can also occur within the scrambled controls (Baek et al., 2014). Thus, this study reveals that shRNA knockdown of DCX and inhibition of DCX expression, does not affect the development of hippocampal NPCs.

Our conclusion leaves us with the question as to why adult-generated dentate NPCs would expend energy to transiently express DCX for no critically functional role? Since DCX is considered a structural protein and is also expressed in non-neurogenic cells, it is possible that DCX is not required for cell survival or neuronal maturation, but may have other roles such as in structural plasticity (Bonfanti and Nacher, 2012). Another possibility is that we observed no phenotype following a knockout of DCX due to compensation by other redundant genes. In support of this hypothesis, double knockout mutants for *Dcx* and *Lissencephaly gene 1* show a severe neuronal migration phenotype that is not observed in single *Dcx* knockout mice (Pramparo et al., 2010). Similarly, double knockout for *Dcx* and *Doublecortin-like kinase* show a severe neuronal migration deficit not observed in the single *Dcx* knockout mice (Koizumi et al., 2006a). Additionally, Doublecortin-like, a splice variant of DCLK, is also expressed in the adult dentate where it localizes with DCX in the immature neuronal population and thus is a possible candidate to also compensate for DCX (Saaltink et al., 2012). This hypothesis may suggest rapid changes in expression of these genes even in the presence of a conditional adult-generated knockout. Future studies are required to determine whether within the developing neurons in the adult dentate there is a genetic interaction that may in part compensate for DCX and contribute to the normal development of adult-generated dentate neurons in the absence of DCX *in vivo*.

### Conflict of interest statement

The authors declare that the research was conducted in the absence of any commercial or financial relationships that could be construed as a potential conflict of interest.

## Supplementary material

Supplementary Figure 1 **Counts of Ctrl-GFP+ and ShDCX-GFP+ cells at 3, 12, and 30 dpi**. There were significantly fewer shDCX-GFP+ cells at 3 and 12 dpi compared to Ctrl-GFP+ cells. *N* = 3–6 mice per group. ^****^*p* < 0.0001, ^*^*p* < 0.02.
