# Supplementary figures and images for: Doublecortin (DCX) is not Essential for Survival and Differentiation of Newborn Neurons in the Adult Mouse Dentate Gyrus
Source: Front Neurosci. 2016 Jan 11;9:494. doi: 10.3389/fnins.2015.00494 (PMC4707254; doi:10.3389/fnins.2015.00494)

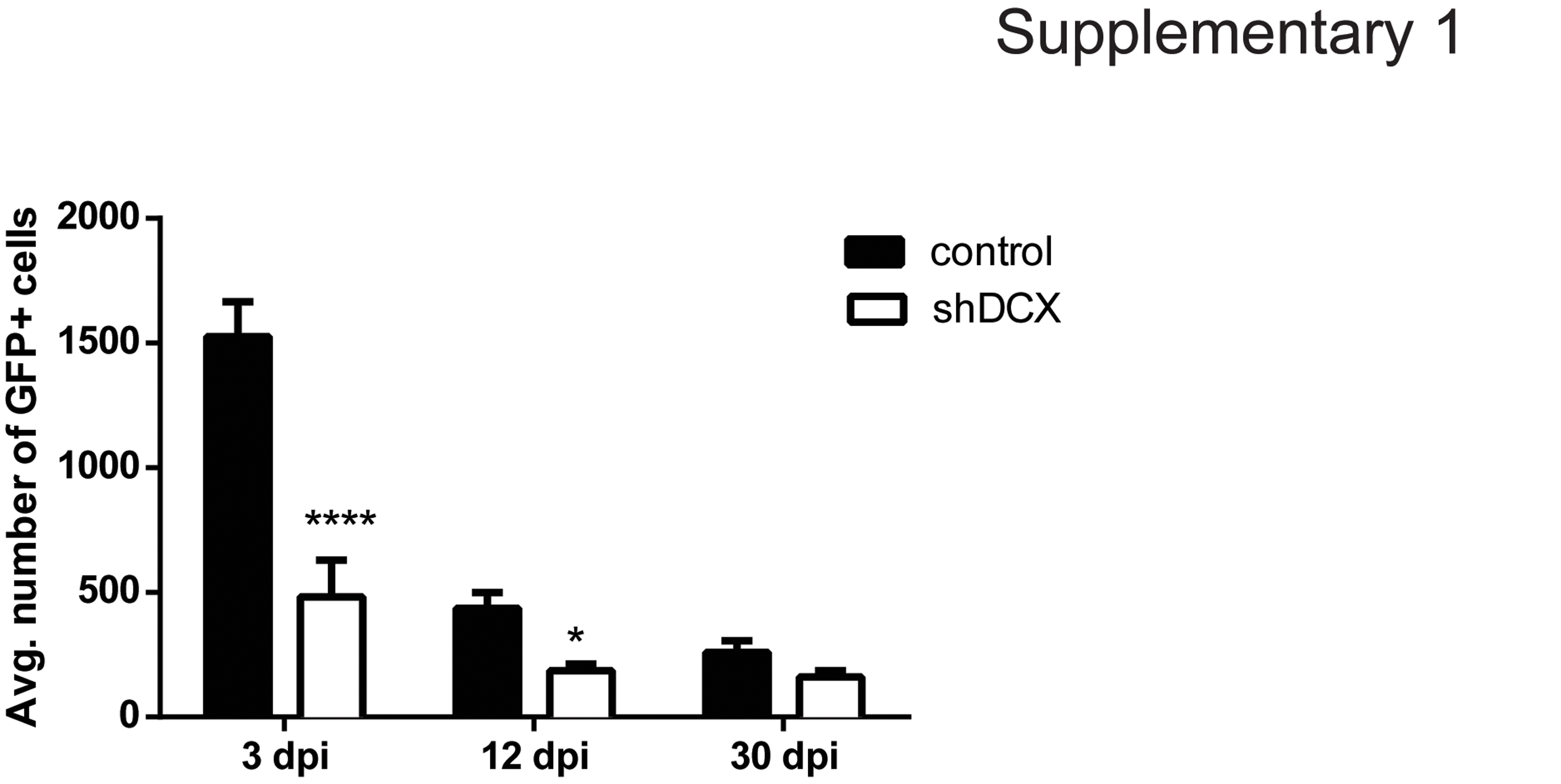

Supplement: Supplementary Figure 1 — Counts of Ctrl-GFP+ and ShDCX-GFP+ cells at 3, 12, and 30 dpi. There were significantly fewer shDCX-GFP+ cells at 3 and 12 dpi compared to Ctrl-GFP+ cells. N = 3–6 mice per group. ****p < 0.0001, *p < 0.02. [file Image1.TIF]
